# Supplementary material for: Resolving the full spectrum of human genome variation using Linked-Reads
Source: Genome Res. 2019 Apr;29(4):635–45. doi: 10.1101/gr.234443.118 (PMC6442396; doi:10.1101/gr.234443.118)
Supplement: Supplemental Material [file supp_gr.234443.118_Supplemental_longranger-2.2.zip › longranger-2.2.tar.bz2!/lr2.2-public/lib/stan/cmdstan/stan/doc/api/html/math_2functions_2primitive__value_8hpp_source.html]

Stan: src/stan/math/functions/primitive\_value.hpp Source File


|  |  |
| --- | --- |
|  | Stan  2.5.0  probability, sampling & optimization |


- Main Page
- Related Pages
- Namespaces
- Classes
- Files

- File List
- File Members

All Classes Namespaces Files Functions Variables Typedefs Enumerations Enumerator Friends Macros Pages

- src
- stan
- math
- functions

primitive\_value.hpp

Go to the documentation of this file.

1 #ifndef STAN\_\_MATH\_\_FUNCTIONS\_\_PRIMITIVE\_VALUE\_HPP

2 #define STAN\_\_MATH\_\_FUNCTIONS\_\_PRIMITIVE\_VALUE\_HPP

3

4 #include <boost/utility/enable\_if.hpp>

5 #include <boost/type\_traits/is\_arithmetic.hpp>

6 #include <stan/math/functions/value\_of.hpp>

7

8 namespace stan {

9

10  namespace math {

11

27  template <typename T>

28  inline

29  typename boost::enable\_if<boost::is\_arithmetic<T>, T>::type

30  primitive\_value(T x) {

31  return x;

32  }

33

44  template <typename T>

45  inline

46  typename boost::disable\_if<boost::is\_arithmetic<T>, double>::type

47  primitive\_value(const T& x) {

48  using stan::math::value\_of;

49  return value\_of(x);

50  }

51

52  }

53

54 }

55

56 #endif

stan::math::value\_of

double value\_of(const T x)

Return the value of the specified scalar argument converted to a double value.

**Definition:** value\_of.hpp:24

stan::math::primitive\_value

boost::enable\_if< boost::is\_arithmetic< T >, T >::type primitive\_value(T x)

Return the value of the specified arithmetic argument unmodified with its own declared type...

**Definition:** primitive\_value.hpp:30

value\_of.hpp


---

[ Stan Home Page ]

*© 2011–2014,
Stan Development Team.*
